# Assessment of knowledge, attitude, and practices towards canine visceral leishmaniasis based on the one health concept in Weliso and Ejaji Towns, Oromia, Ethiopia

**DOI:** 10.1038/s41598-023-47340-0

**Published:** 2023-11-25

**Authors:** Weraka Weya Diriba, Endrias Zewdu Gebremedhin

**Affiliations:** 1 Department of Resource Development and Income Generation Directorate, Ambo University, Main Campus, P. O. Box 19, Ambo, Ethiopia; 2 School of Veterinary Medicine, Department of Veterinary Sciences, Ambo University, Mamo Mezemir Campus, P. O. Box 19, Ambo, Ethiopia

**Keywords:** Diseases, Risk factors

## Abstract

Canine visceral leishmaniasis (CVL) is a significant vector-borne Meta zoonotic disease caused by agents of the *L. donovani* complex. The disease is transmitted by the bite of *phlebotomine* female sandflies of the genera *Phlebotomus* and *Lutzomyia* in the old and new worlds, respectively. This study was conducted to assess the knowledge, attitude, and practices of the residents about CVL based on the One Health concept in two towns of the Oromia Region, Ethiopia. A community-based cross-sectional study was conducted between October 2019 and September 2020, using an interview questionnaire as the study instrument. The study participants were selected through a simple random sampling method. Pearson’s Chi-square and logistic regression tests were used to evaluate the association between the study participants’ knowledge, attitude, and practices toward CVL and possible risk factors. The study included a total of 400 participants, and the results indicated that 77.25% had good knowledge, 60.5% had a favorable attitude, and 59.25% had good practices toward CVL. The town of Ejaji and dog ownership were significantly associated with good knowledge (*p* = 0.001), and attitude (*p* = 0.001) towards CVL, while having a dog (*p* = 0.001), having a diploma (*p* = 0.019) or degree and above (0.018), being divorced or widowed (0.012), and being Oromo (*p* = 0.013) were all significantly associated with good CVL practice. Most participants in both study areas had good knowledge but moderate attitudes and practices toward CVL. Therefore, it is crucial to undertake comprehensive community health education and awareness programs of zoonotic visceral leishmaniasis and its vectors based on the One Health concept through various means.

## Introduction

Canine visceral leishmaniasis (CVL) is a major vector-borne meta-zoonotic disease caused by an obligate intramacrophage protozoon of the genus *Leishmania* in the family Trypanosomatidae^1^. The etiological agents of CVL belong to the *Leishmania donovani* complex: *L. d. donovani*, *L. d. infantum*, and *L. d. archibaldi* in the old world and *L. d. chagasi* in the new world. Leishmaniasis is transmitted by the bite of *phlebotomine* female sandflies of the genera *Phlebot-omus* and *Lutzomyia* in the old and new worlds, respectively^2^. There are three forms of leish-maniasis, visceral (VL), cutaneous (CL), and mucocutaneous (MCL). Of the three forms, VL is the most prevalent in eastern Africa, followed by CL and MCL^3^.

The parasite plays great medical and veterinary public health significance as it infects numerous mammal species, including humans. The primary reservoir hosts of *Leishmania* species are sylv-atic mammals such as forest rodents, hyraxes, and wild canids. Among domesticated animals, dogs are the most important species in the epidemiology of this disease. Leishmaniasis is a neglected tropical disease (NTD). About 53 species of the parasite have been described from different regions of the world; out of these, 31 species are known to be parasites of mammals and 20 species are pathogenic to human beings^4^.

Canine visceral leishmaniasis is mainly caused by *L. infantum* and is typically characterized by a chronic and multisystemic nature. The primary mode of transmission is through the bite of sandflies carrying the infection. It can result in a range of symptoms, such as pyrexia, anemia, weight loss, alopecia, exudative dermatitis, hyperkeratosis of the footpad, onychogryphosis, pale mucous membrane, generalized lymph adenomegaly, malaise, cachexia, epistaxis, pancytopenia, hypergammaglobulinemia, hepatosplenomegaly, granulomatous type of inflammation shifting to lameness, chronic renal failure, progressive suppression of the cellular immune response, weak-ness, and hematological alteration leading to fatality^5–7^.

A confirmed diagnosis can be achieved with parasitological, serologic, or molecular tests^8^. Unfortunately, many dog owners only seek veterinary assistance when the disease has reached an advanced stage^9^. The treatment of CVL can be costly and complex, posing significant constraints in many cases. As a result, veterinarians and dog owners are often faced with the difficult decision of opting for euthanasia as a means to alleviate the suffering of the affected dogs^10^. The occurrence of severe forms of CVL characterized by emaciation and skin lesions has likely had a significant impact on dog owners, leading to negative memories and contributing to their knowledge about the disease^10,11^.

Leishmaniasis is a significant disease that affects all continents except Oceania, putting about 350 million people at risk of contracting it^12^. The number of VL cases in humans is expected to be over 500,000 each year, with most cases occurring in South America, East Africa, and the Indian subcontinent. Over 90% of all cases of VL in the world are found in Bangladesh, Brazil, Ethiopia, India, Nepal, and Sudan. The second-highest number of VL cases, after the Indian Subcontinent, is found in Eastern Africa. Eritrea, Ethiopia, Kenya, Somalia, North Sudan, Southern Sudan, and Uganda all have high rates of the illness^5^.

Ethiopia has been identified as one of the top ten countries with the highest estimated numbers of VL cases, accounting for 70–75% of the global incidence of the disease. However, out of an estimated 2 million occurrences, only roughly 600,000 are known to have been recorded^13^. According to Kebede^14^, the epidemiology of VL in Ethiopia has been changing, with endemic areas continually expanding. This shift may be attributed to the current trend of climate change, rapid urbanization, and massive population movements, affecting the range and population density of insect vectors and reservoir hosts and leading to a cumulative rise in the rate of human infection^15^.

Numerous investigations have unequivocally shown that VL occurs in many parts of Ethiopia. Cases of VL have been reported from six regions (Tigray, Amhara, Oromia, Southern Nations Nationalities, and People’s Region (SNNPR), Somali and Afar Regional States), The Metema and Humera plains, Welkayit Tsegede, Gibdo, Raya and Kobo in North and Northwest Ethiopia, the Omo plains, the Aba Roba plains, and the Woito River Valley in SNNPR are the most significant endemic foci. Additionally, the Afder and Liben Zones in the Ethiopian Somali Region, the Moyale area and Genale river basin in the Oromia Regional State, and the Awash Valley in the Afar Regional State have all recorded cases of the disease^16^. Besides, serologically positive cases of humans and dogs were reported from Gambella and Benishangul-Gumuz regional states^17^, and a recent study has also unveiled CVL seropositive dogs in Ambo, Weliso, Ejaji, Gojo, and Bako towns of West and Southwest Shewa Zones, Oromia, Ethiopia^18,19^.

The community's involvement is the most crucial requirement for the success of any disease prevention and control initiatives. As the key factors of community engagement, disease-related KAP in the community must be understood by program implementers. However, there have been few dependent research outputs on the KAP of the community toward CVL in Ethiopia, and many studies have been done on human visceral leishmaniasis (HVL) in specific regions of the country. Therefore, the aim of this study was to assess the KAP of the residents toward CVL based on the One Health concept in the towns of Weliso in the Southwest Shewa Zone and Ejaji in the West Shewa Zone of Oromia, Ethiopia.

## Material and methods

### Study area

**Figure 1:**
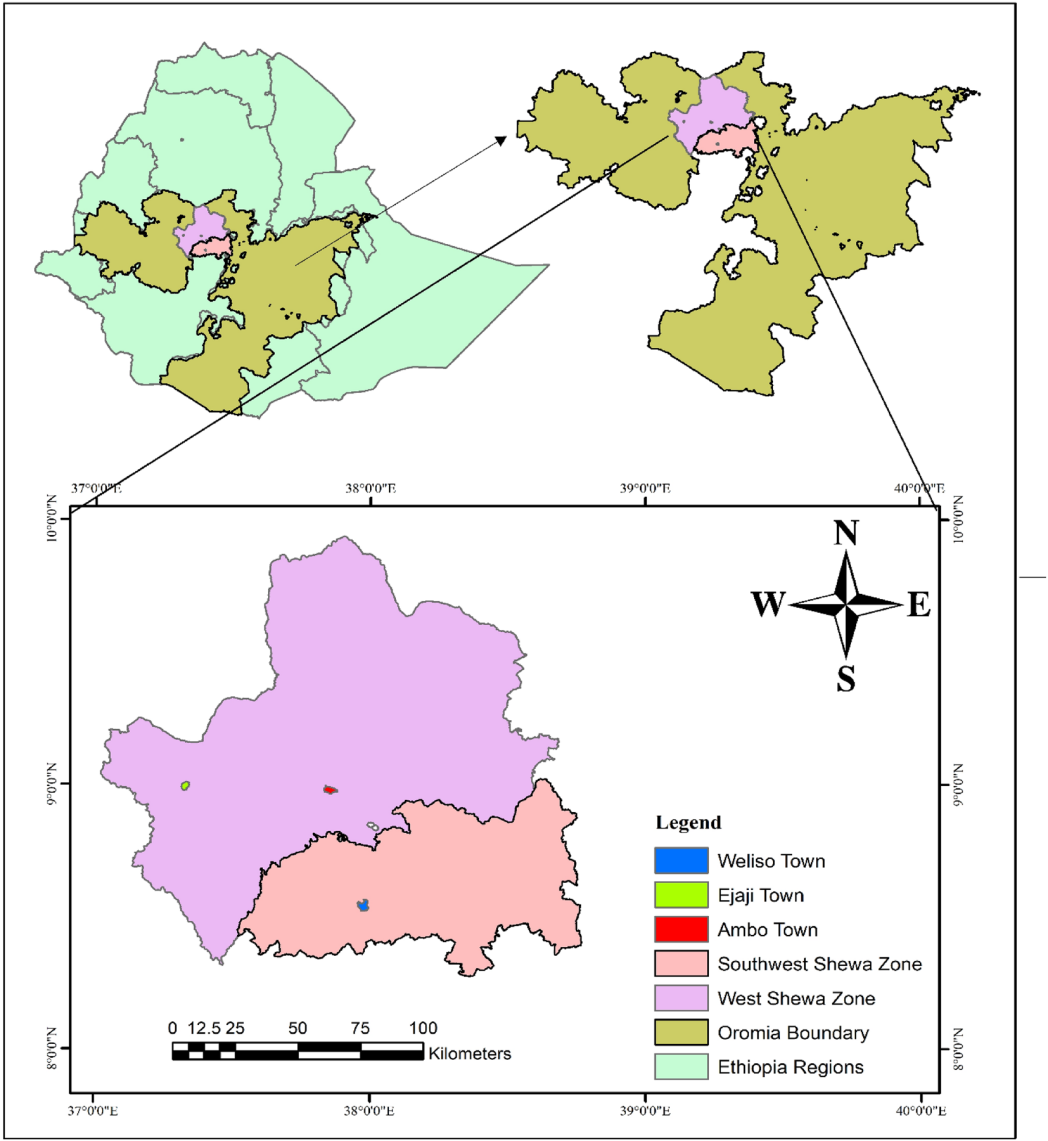
Map showing the current study areas. The map was made using shapefile data of sampling places using ArcGIS 10.4 for Desktop, version 10.4.5524, http://www.esri.com.

The research was undertaken in Weliso town of Southwest Shewa Zone and Ejaji town of West Shewa Zone, Oromia, Ethiopia. Weliso is one of the 14 Districts comprising the Southwest Shewa Zone. Weliso town is the administrative center of the Weliso District, which has a Hospital, Health Centers, College, Secondary (Preparatory) School, Technique School, and other facilities. It is located 114 km Southwest of Addis Ababa, with a longitude of 37° 57ʹ 59.99″ E and a latitude of 8° 31ʹ 59.99″ N, with an elevation of 2063 m above sea level (m.a.s.l). The annual rainfall and temperatures are 950–2718.3 mm and 13.6–25 °C, respectively. Ejaji town, on the other hand, is the administrative center of the Elu Gelan District, which is 90 km West of the Zonal headquarters, Ambo, and 215 km West of Addis Ababa. Ejaji town has a Health Center and Secondary School and is located at a latitude of 8° 59.9 ʹN and a longitude of 37° 9.8 ʹE, with an altitude of 1565–1790 m.a.s.l. The average annual rainfall and temperatures are 2000–2300 mm and 27–30 °C, respectively. The research locations’ climatic characteristics range from extremely hot (lowland) through temperate (middle) to cold temperatures (highland)^20^ (Fig. 1).

### Study population

The household heads in the current study areas comprise the study population. Weliso town had a total population of 37,798 people as per the CSA^20^ count, of which 18,800 (49.84%) were males while the other 18,998 (50.16%) were female, and 2546 household heads made up the population. There were 3969 household heads in Ejaji Town, which had a total population of 18,740 people, of which 9741 (51.98%) were males and 8999 (48.02%) were females. Participants in the study were divided into three residency categories: newcomers (those who had only been in the study locations for six months to five years), residents (those who had lived there for six to twenty years), and natives (those who had lived there for more than twenty years).

### Study design

A community-based cross-sectional study was conducted from October 2019 to September 2020 among residents of Weliso Town of Southwest Shewa Zone and Ejaji Town of West Shewa Zone, Oromia, Ethiopia, using an interview questionnaire as the study instrument, aiming to assess the KAP of the residents toward CVL based on the One Health concept. Canine visceral leishmaniasis is a chronic and potentially fatal protozoan zoonotic disease of particularly the dog's viscera (liver, spleen, bone marrow, and lymph nodes). Human visceral leishmaniasis is a chronic and potentially fatal protozoan zoonotic disease of particularly the human viscera (liver, spleen, bone marrow, and lymph nodes). These diseases are commonly caused by *L. donovani* complex infection and transmitted by the bite of an infected *phlebotomine* female sandfly. The study participants were selected using a simple random sampling technique. This involves selecting “n” people at random from a sample frame list of “N” people. Only one family member who was older than 18 years old from each household participated in this study. The researcher interviewed the household heads at various public meetings to get the targeted household heads and also conducted home visits with each participant to collect all necessary data on the questionnaire survey, including demographic characteristics, knowledge (8 questions), attitude (6 questions), and practice (6 questions).

### Inclusion and exclusion criteria

The household head’s agreement to take part in the study was a requirement for inclusion. Individuals over the age of 18 who had been in the study areas for six months were included in the study in order to evaluate the KAP questions; however, those who were seriously ill or unable to respond were eliminated from the study, and the houses before and after the indicated one were sampled for replacement.

### Sample size determination

To conduct more comprehensive evaluations of state effects, such as multiple regressions, covariance analyses, or log-linear analyses, a large sample size is necessary. Therefore, the required sample size for the study was created as recommended by Israel^21^. Consequently, a 400-household heads sample size was created in order to evaluate the KAP of the community toward CVL in the municipalities of Weliso and Ejaji, with 50% of the sample coming from each town (200/400)^21^.

### Data collection

A standardized questionnaire consisting of 20 open-ended and closed-ended questions was used to collect data on demographic characteristics and KAP related to CVL. The questionnaire was meticulously designed, pretested, and assessed, comprising eight questions focusing on knowledge, six on attitudes, and six on practices related to CVL. The questionnaire was originally written in English and then translated into local languages (Afan Oromo and Amharic) authorized and pre-tested before the actual data collection. To ensure consistency, the questionnaire was pretested on 20 household heads.

### Scoring

On the basis of the methods outlined by Alemu et al.^22^, the KAP of the respondents was rated. Each accurate response received a knowledge score of 1, while any inexact or unsure response received a score of 0. Scores for general knowledge can range from 0 to 8. Scores between 0 and 4 on the Knowledge scale were regarded as poor knowledge, while scores over 4 were regarded as good knowledge. A 6-item survey was used to assess attitudes toward the CVL. Scores of 0–3 were regarded as unfavorable attitudes, while scores of 3 or more were regarded as favorable attitudes. A 6-item questionnaire was used to evaluate the practice, and answers between 0 and 3 were regarded as poor practice and those between 4 and 8 as good practice. Additionally, comments that agreed and strongly agreed received one mark, whereas responses with disagree and strongly disagree got zero marks.

### Data management and analysis

The Microsoft Excel 2019 spreadsheet was used to generate a database from the raw data collected from the questionnaire survey, which was then refined and coded. The STATA version 14 software (Stata Corp, College Station, USA) was used to analyze the obtained data. We compiled the data using descriptive statistics. The association between the risk factors and the respondents’ KAP was assessed using Pearson's Chi-square (${x}^{2}$) and logistic regression (univariable and multivariable) tests.

Strongly agreeing and agreeing responses to the KAP questionnaire survey were merged to form the word “agree,” whereas strongly disagreeing and disagreeing responses were merged to form the word “disagree.” Small frequency distribution results from risk factors like age (36–50 and > 50 years), occupation (student and others; housewife, priest), religion (Muslims and Wakefeta (Oromo's cultural democratic administration system)), marital status (divorced and widowed), ethnicity (Gurage, Amhara, and Tigre), and year of residency (resident and newcomer) were merged. The recorded statistics were arranged in ascending order, starting at the prevalence with the lowest value.

The socio-demographic traits of the household heads were used as hypothesized independent variables in a separate analysis of the KAP (dependent variables). In addition, the independent variables’ collinearity (confounding) was examined. From the collinear independent variables, only one independent variable with a substantial connection with the result was chosen and used for the logistic regression model’s final analysis. After examining for multicollinearity, the variables having a *p* value ≤ 0.25 in the univariable logistic regression analysis were further examined using multivariable logistic regression. In all the cases *p* < 0.05 was set for significance.

### Ethical approval

The study was approved by the ethics committee of Ambo University, Mamo Mezemir Campus, College of Agriculture and Veterinary Sciences, Ethiopia, and written permission was acquired from the concerned body of Ambo University before data collection. All study participants were informed about the study objective as per the recommendation of Ambo University’s research ethical guidelines. The questionnaire was administered to the head or members of households after getting verbal consent to participate because some of the participants did not read and write. All participants’ full cooperation and voluntary participation (verbal consent) were acquired by assuring them of the anonymity of their involvement. The verbal consent procedure was approved by the ethics committee of Ambo University, Mamo Mezemir Campus, College of Agriculture and Veterinary Sciences.

## Results

### Study participants

The current KAP study had 400 research participants in total. Of this 50% (200/400) are from Weliso and 50% (200/400) are from Ejaji towns. Of the 400 participants, 50% (200/400) were dog owners, and the rest 50% (200/400) were non-dog owners. According to the findings of the current study, the male participants, young adult age groups, those with secondary education, government employees, and individuals following Protestant and Orthodox Christianity were higher in both towns. In addition, the majority of the respondents in both towns were married and as the study was conducted in the Oromia Regional State, the Oromo ethnic group was dominant with most respondents being natives of the region (Table 1).

**Table 1:** Frequency distribution.

Variables	Category	Frequency	%
Town	Weliso	200	50
	Ejaji	200	50
Dog ownership	No	200	50
	Yes	200	50
Sex	Female	52	13
	Male	348	87
Age in years	> 50	24	6
	36–50	99	24.75
	18–35	277	69.25
Education	MSc	3	0.75
	Illiterate	29	7.25
	Diploma	60	15
	Primary	82	20.5
	Degree	102	25.5
	Secondary	124	31
Occupation	*Others	17	4.25
	Student	52	13
	Farmers	94	23.5
	Self-employee	104	26
	Gov. employee	133	33.25
Religion	Wakefeta	6	1.5
	Muslim	22	5.5
	Orthodox	155	38.75
	Protestant	217	54.25
Marital status	Widowed	3	0.75
	Divorced	6	1.5
	Single	124	31
	Married	267	66.75
Ethnicity	Tigre	4	1
	Gurage	24	6
	Amhara	25	6.25
	Oromo	347	86.75
Year of residency	Newcomer	17	4.25
	Resident	34	8.5
	Native	349	87.25

*Others = housewife, priest, MSc = Masters.

### Knowledge of the respondents

The current study revealed 77.25% (309/400) overall good knowledge. Of these 62.5% (125/200) in Weliso, and 92% (184/200) in Ejaji towns had good knowledge of CVL (Table 2).

**Table 2:** Results of the computed knowledge.

Variables	Category	Frequency	%
Heard about CVL?	No	64	16
	Yes	336	84
Causative agent?	Others/monkey meat	3	0.75
	The genus *Salmonella*	4	1
	The genus *Brucella*	5	1.25
	The genus *Leptospira*	12	3
	The genus *Leishmania*	167	41.75
	I don`t know	209	52.25
Is CVL a vector-borne zoonosis?	Disagree	2	0.5
	Neutral	76	19
	Agree	322	80.5
Which vector transmits the disease?	Culicoides mosquitoes	20	5
	Anopheles mosquitoes	47	11.75
	Stomoxys flies	50	12.5
	*Others	64	16
	I don`t know	87	21.75
	Sand flies	132	33
Primary reservoir host for CVL?	Elephants, buffaloes, giraffe rhinoceros, and deer	1	0.25
	*Others	1	0.25
	Wild birds like pigeons, bats	15	3.75
	Domesticated animals like equines, cattle, sheep, goats, and pigs	28	7
	I don`t know	70	17.5
	Sylvatic canids and domesticated dogs	285	71.25
Clinical signs observed in dogs?	Diarrhea and coughing	9	2.25
	Lameness and blindness	10	2.5
	I don’t know	77	19.25
	Ulcerations, alopecia, desquamation of skin, generalized nodular lesions or pustules	304	76
If CVL is left untreated what will be the outcome?	Self-cure	34	8.5
	I don’t know	96	24
	100% fatal	270	67.5
Is CVL a preventable disease?	Disagree	6	1.5
	Neutral	73	18.25
	Agree	321	80.25
Results of good knowledge	Weliso	125	62.5
	Ejaji	184	92
	Total	309	77.25

*Others = monkey meat, spleen, contact, *others = monkey, CVL = Canine Visceral Leishmani-asis, VL = Visceral leishmaniasis.

All the independent variables considered in this study toward knowledge of CVL were non-collinear. Moreover, all *p* values of the independent variables such as town, dog ownership, sex, ethnicity, and year of residency were $\le$ 0.25. Hence, all were selected for the final model. The current study revealed that Ejaji town (*p* = 0.001) and dog ownership (*p* = 0.001) were strongly associated with good knowledge. The likelihood of Ejaji town respondents' good knowledge was (AOR = 7.79) and dog owners (AOR = 5.04) times higher than Weliso town and non-dog owners (Table 3).

**Table 3:** Univariable and multivariable logistic regression analysis results of the computed knowledge.

Variable	Category	No. tested	No. Good Knowledge (%)	Univariable		Multivariable	
				COR (95% CI)	*p* Value	AOR (95% CI)	*p* Value
Town	Weliso	200	125(62.5)	1	1	1	1
	Ejaji	200	184(92)	6.9(3.84–12.39)	0.001*	7.79(4.14–14.68)	0.001*
Dog ownership	No	200	131(65.5)	1	1	1	1
	Yes	200	178(89)	4.26(2.51–7.24)	0.001*	5.04(2.8–9.06)	0.001*
Sex	Female	52	36(69.23)	1		1	
	Male	348	273(78.45)	1.62(0.85–3.07)	0.142	1.14(0.54–2.41)	0.736
Age	18–35	276	212(76.81)	1	1	–	–
	> 35	124	97(78.23)	1.08(0.65–1.81)	0.755	–	–
Education	Secondary	124	82(66.13)	1	1	–	–
	Primary	82	60(73.17)	1.4(0.76–2.58)	0.286	–	–
	Diploma	60	45(75)	1.54(0.77–3.07)	0.224	–	–
	Degree and above	105	94(89.52)	4.38(2.12–9.05)	0.001*	–	–
	No formal education	29	28(96.55)	14.34(1.89–109.1)	0.010*	–	–
Occupation	Self-employee	104	58(55.77)	1	1	–	–
	Students AND *others	69	41(59.42)	1.16(0.63–2.15)	0.635	–	–
	Gov. employee	133	123(92.48)	9.76(4.6–20.69)	0.001*	–	–
	Farmer	94	87(92.55)	9.86(4.16–23.34)	0.001*	–	–
Religion	Orthodox	155	96(61.94)	1	1	–	–
	Muslim and Wakefeta	28	19(67.86)	1.3(0.55–3.06)	0.551	–	–
	Protestant	217	194(89.4)	5.18(3.02–8.9)	0.001*	–	–
Marital status	Divorced and widowed	9	6(66.67)	1	1	–	–
	Single	124	85(68.55)	1.09(0.26–4.58)	0.907	–	–
	Married	267	218(81.65)	2.22(0.54–9.2)	0.270	–	–
Ethnicity	Gurage, Amhara, Tigre	53	34(64.15)	1	1	1	1
	Oromo	347	275(79.25)	2.13(1.15–3.96)	0.016*	1.49(0.68–3.28)	0.320
Year of residency	Native	349	266(76.22)	1	1	1	1
	Resident and Newcomer	51	43(84.31)	1.68(0.76–3.71)	0.202	1.54(0.58–4.1)	0.383

No. = Number, *others = Housewife, priest, COR = crude odd ratio, AOR = adjusted odd ratio.

### Attitudes of the respondents

The current study revealed 60.5% (242/400) overall favorable attitude. Of these 49.5% (99/200) in Weliso, and 71.5% (143/200) in Ejaji towns had a positive attitude (Table 4).

**Table 4:** Results of the attitude.

Variables	Category	Frequency	%
Is CVL a health problem in dogs in the study areas?	No	3	0.75
	I don’t know	84	21
	Yes	313	78.25
Contract any form of leishmaniasis?	No	8	2
	I don’t know	67	16.75
	Yes	325	81.25
Is CVL a curable disease?	I don’t know	37	9.25
	No	75	18.75
	Yes	288	72
Preferred medication to treat CVL?	*Others	32	8
	Traditional healer	55	13.75
	I don’t know	72	18
	Health center and traditional healer	73	18.25
	Health center	168	42
How do you care for CVL in dogs?	Precaution in diet	5	1.25
	Cleanliness	47	11.75
	Anti-leishmanial drugs and dog collars	48	12
	Cleanliness, isolation of the patient, precaution in diet	58	14.5
	Isolation of the patient	59	14.75
	*Others	77	19.25
	I don’t know	106	26.5
Is there community participation to prevent and control CVL?	I don’t know	5	1.25
	Yes	58	14.5
	No	337	84.25
Results of positive attitude	Weliso	99	49.5
	Ejaji	143	71.5
	Total	242	60.5

*Others = self-cure, not cure and 100% fatal, *others = killing, medication for leishmaniasis is not available in veterinary clinics in Ethiopia.

All the independent variables considered in this study towards the attitude of CVL were non-collinear except occupation and education (*r* = 0.5442). Among collinear independent variables, none were selected for the final model since their p-value was greater than or equal to 0.25. However, town, dog ownership, ethnicity, and year of residency were selected for the final model because their *p* values were $\le$ 0.25. The final model revealed that Ejaji town (*p* = 0.001) and dog ownership (*p* = 0.001) were significantly associated with positive attitudes. The likelihood of Ejaji town respondents' positive attitudes was (AOR = 2.65) and dog owners (AOR = 23.4) times higher than Weliso town and non-dog owners (Table 5).

**Table 5:** Univariable and multivariable logistic regression analysis results of the attitudes.

Variable	Category	No. tested	No. Pos. attitude (%)	Univariable		Multivariable	
				COR (95% CI)	*p* Value	AOR (95% CI)	*p* Value
Towns	Weliso	200	99(49.5)	1	1	1	1
	Ejaji	200	143(71.5)	2.56(1.69–3.87)	0.001*	2.65(1.67–4.21)	0.001*
Dog ownership	No	200	88 (44.)	1	1	1	1
	Yes	200	154 (77.)	4.26(2.77–6.56)	0.001*	23.4(2.86–7.25)	0.001*
Sex	Female	52	31(59.62)	1	1	–	–
	Male	348	211(60.63)	1.29(0.72–2.32)	0.396	–	–
Age	> 35	124	70(56.91)	1	1	–	–
	18–35	276	172(62.09)	1.18(0.77–1.81)	0.445	–	–
Education	Secondary	124	60(48.39)	1	1	–	–
	Primary	82	46(56.1)	1.71(0.97–3)	0.061	–	–
	No formal education	29	17(58.62)	2.89(1.5–5.54)	0.001*	–	–
	Diploma	60	38(63.33)	4.52(2.54–8.06)	0.001*	–	–
	Degree and MSc	105	81(77.14)	1.65(0.73–3.72)	0.228	–	–
Occupation	Self-employee	104	45(43.27)	1	1	–	–
	Students and *others	69	30(43.48)	1.56(0.84–8.32)	0.978	–	–
	Farmer	94	60(63.83)	2.31(1.3–4.1)	0.004*	–	–
	Gov. employee	133	107(80.45)	5.39(3.03–9.62)	0.001*	–	–
Religion	Orthodox	155	71(45.81)	1	1	–	–
	Muslim and Wakefeta	28	15(53.57)	1.23(0.55–2.76)	0.614		
	Protestant	217	156(71.89)	2.24(1.46–3.42)	0.001*	–	–
Marital status	Single	124	65 (52.42)	1	1	–	–
	Married	267	171(64.04)	1.67(1.09–2.57)	0.020*	–	–
	Divorced and widowed	9	6(66.67)	7.1(0.97–65.88)	0.053	–	–
Ethnicity	Gurage, Tigre, and Amhara	53	25(47.17)	1	1	1	1
	Oromo	347	217(62.54)	1.61(0.9–2.88)	0.107	2.01(0.95–4.25)	0.067
Year of residency	Native	349	205(58.74)	1	1	1	1
	Resident and Newcomer	34	22(64.71)	1.59(0.85–2.94)	0.147	1.66(0.74–3.73)	0.218

No. = Number, *others = Housewife, priest, Pos. = positive, COR = crude odd ratio, AOR = adjusted odd ratio.

### Practices of the respondents

The current study revealed 59.25% (237/400) overall good practice. Of these 59% (118/200) in Weliso, and 59.5% (119/200) in Ejaji towns had good practices (Table 6).

**Table 6:** Results of the practice.

Variables	Category	Frequency	%
The habit of sleeping on the ground floor or outside of the home	I don’t know	4	1
	No	47	11.75
	Yes	349	87.25
If yes, where?	In the animal’s corral or pen	20	5.73
	Temporary home at the farm	23	6.59
	Under tree shade	83	23.78
	Under tree shade, in the animal’s corral or pen, and a temporary home at the farm	223	63.89
Use repellents in humid seasons	No	154	38.5
	Yes	246	61.5
If yes, what type of repellents did you use?	*Others	5	2
	Malathion, canned insecticide, and natural wood	34	13.82
	Malathion	39	15.85
	Canned insecticide	56	22.76
	Natural wood	112	45.53
Do you clean the dogs/your house routinely?	No	142	35.5
	Yes	258	64.5
Can CVL be controlled?	Disagree	11	2.75
	Neutral	114	28.5
	Agree	275	68.75
Results of good practice	Weliso	118	59
	Ejaji	119	59.5
	Total	237	59.25

*Others = dung burning, powder insecticide.

All the independent variables considered in this study toward the practice of CVL were non-collinear. Thus, none of them were selected for the final model. However, dog ownership (*p* = 0.001), education (*p* = 0.228, 0.061, 0.001, and 0.001), marital status (*p* = 0.020 and 0.053), ethnicity (*p* = 0.017), and year of residency (*p* = 0.147) were selected for the final model (*p* values $\le$ 0.25). Also, dog ownership (*p* = 0.001), education (diploma and degree and above) (*p* = 0.019 and 0.018), marital status (divorced and widowed) (0.012), and ethnicity (Oromo) (*p* = 0.013) were strongly associated with good practice. The likelihood of dog owners' good practice was (AOR = 23.42), education level of (diploma and degree, and above) (AOR = 2.68 and 2.51) respectively, marital status of (divorced and widowed) (AOR = 4.8), and ethnicity of (Oromo) (AOR = 3.11) times higher than non-dog owners, education level of the secondary, marital status of single, and ethnicity of Gurage, Amhara, and Tigre (Table 7).

**Table 7:** Univariable and multivariable logistic regression analysis results of the practices.

Variable	Category	No. tested	Good practice no. (%)	Univariable		Multivariable	
				COR (95% CI)	*p* Value	AOR (95% CI)	*P* Value
Towns	Weliso	200	118(59)	1	1	1	**–**
	Ejaji	200	119(59.5)	1.02(0.68**–**1.52)	0.919	**–**	**–**
Dog ownership	No	200	60(30)	1	1	1	1
	Yes	200	177(88.5)	17.96(10.58**–**30.5)	0.001*	23.42(12.53**–**43.8)	0.001*
Sex	Female	52	28(53.85)	1	1	**–**	**–**
	Male	348	209(60.06)	1.29(0.72**–**2.31)	0.396	**–**	**–**
Age	> 35	124	70(56.91)	1	1	**–**	**–**
	18**–**35	276	167(60.29)	1.18(0.77**–**1.81)	0.445	**–**	**–**
Education	Secondary	124	53(42.74)	1	1	1	1
	No formal education	29	16(55.17)	1.64(0.73**–**3.72)	0.228	1.25(0.38**–**15.91)	0.720
	Primary	82	46(56.10)	1.71(0.97**–**3.00)	0.061	1.43(0.66**–**3.08)	0.367
	Diploma	60	41(68.33)	2.89(1.51**–**5.54)	0.001*	2.68(1.18**–**6.12)	0.019*
	Degree and above	105	81(77.14)	4.52(2.53**–**8.05)	0.001*	2.51(1.17**–**5.4)	0.018*
Occupation	Students and *others	69	27(39.13)	1	1	**–**	**–**
	Self**-**employee	104	52(50.00)	1.56(0.84**–**2.88)	0.161	**–**	**–**
	Farmer	94	53(56.38)	3.75(2.13**–**6.61)	0.001*	**–**	**–**
	Gov. employee	133	105(78.95)	1.29(0.74**–**2.26)	0.369	**–**	**–**
Religion	Orthodox	155	75(48.39)	1	1	**–**	**–**
	Muslim and Wakefeta	28	15(53.57)	1.23(0.55**–**2.76)	0.614	**–**	**–**
	Protestant	217	147(67.74)	2.24(1.46**–**3.42)	0.001*	**–**	**–**
Marital status	Single	124	62(50.00)	1	1	1	1
	Married	267	167(62.55)	1.67(1.08**–**2.56)	0.020*	1.2(0.64**–**2.26)	0.569
	Divorced and widowed	9	8(88.89)	8.0(0.97**–**65.88)	0.053	24.8(2.03**–**302.77)	0.012*
Ethnicity	Gurage, Amhara, and Tigre	53	26(49.06)	1	1	1	1
	Oromo	347	211(60.81)	1.61(0.90**–**2.88)	0.107	3.11(1.27**–**7.6)	0.013*
Year of residency	Native	349	202(57.88)	1	1	1	1
	Resident and Newcomer	51	35(68.63)	1.59(0.85**–**2.98)	0.147	2.12(0.8**–**5.57)	0.128

No. = Number, *others = Housewife, priest, COR = crude odd ratio, AOR = adjusted odd ratio.

## Discussion

### Knowledge of the respondents

The results of the current investigation showed that 84% of all participants had heard of CVL. The outcome is better than research done in Tunisia, where 39.5% of the participants confirmed knowing CVL^10^. However, it is in line with research done in Addis Zemen Town, Northwest Ethiopia, where 87.4% of candidates have heard of VL^22^; but it is lower than research done in Welkait District, Western Tigray, Ethiopia, where 100% of the interviewed participants have heard of the disease^23^; in Libo Kemkem District, Northwest Ethiopia, where 97.7% of participants had heard about VL^24^; in Armachiho District, Northwestern Ethiopia, where 100% of the interviewed participants had heard of VL^25^, and research done in East Africa (Kenya and Uganda), where 95% participants have heard of VL^26^. This might be due to differences in research and surveillance activities focused on CVL and HVL. Hence, too many similar studies have been done in the northwestern parts of Ethiopia than the current study areas. Besides, participants live in highly endemic areas, and dog ownership has been supposed to have more information about CVL. However, it impacted the existing information gap between the study areas mentioned above.

According to Singh^27^, research done in India found that 73% of participants were aware of the causes of VL, however, in the current study, only 41.75% of participants said that the genus *Leishmania* was the disease's causal agent. Rates of 80.5% of participants surveyed in this study were aware that CVL is a zoonosis spread by vectors. This outcome was better than research done in Tunisia, where 67% of the respondents were aware that CVL is a zoonotic disease^10^; Welkait District, Western Tigray, Ethiopia, where the majority of respondents, 89.02%, were unaware of the significance of VL in zoonotic transmission^23^. Moreover, 33% of participants were aware that the sandfly is the CVL vector. Compared to research done in Tunisia; Welkait District, Western Tigray, Ethiopia; West Armachiho District; and Addis Zemen Town, Northwest Ethiopia, where 48%, 47.1%, 52.1%, and 68% of participants, respectively, understood that the disease's causative agent was transmitted through sandfly bites^10,22,23,25^ the current result is lower.

However, it was better than the rate in Sudan, where just 6% of participants said sandfly bites cause the disease to be transmitted^28^. When we take a look at the comparison of the above results about ZVL, the basic differences seem to have arisen from the lack of awareness made by Veterinary and Medical professionals of different regions about the disease in a sense of the One Health concept.

In the current study, 71.25% of participants were aware that domestic dogs and sylvatic canids serve as the primary reservoir hosts for CVL. This finding is better than research done in Northwest Ethiopia, where 10% of participants thought bats were to blame for the disease^29^. Furthermore, 76% of respondents were aware of multiple symptoms and signs of the illness. The outcome is better than research done in Addis Zemen town, Northwest Ethiopia, where 62% of the participants were aware of more than one sickness indication or symptom^22^. However, this figure is lower than that of research done in the rural communities of Amhara State, Northwest Ethiopia; Welkait District, Western Tigray, Ethiopia; and West Armachiho District, Northwest Ethiopia, where 84.7%, 87.5%, and 88.2% of the participants, respectively, knew more than one signs and symptoms of VL^23,25,30^, as well as results from other rural communities of Nepal and India, in which it raises to 85%^31,32^. Additionally, the main differences between the current study results and the previous ones have been encountered due to the lack of the One Health concept in the ZVL assessment.

In the current study, 67.5% of participants were aware that the outcome in the case of CVL would be 100% deadly if untreated. This finding differs from one from Addis Zemen Town, Northwestern Ethiopia, where 96.7% of participants were aware that if the illness went untreated, death would be the result^22^. Moreover, 80.25% of participants believed that the disease could be prevented, which is in accordance with research done in Tunisia, where 81% of the questioned persons knew that CVL is a preventable disease^10^. However, it is lower than the results of the studies conducted in other Northwest Ethiopian Districts. Specifically, the percentage of participants who knew that VL is preventable was lower in the Districts of Metema, West Armachiho (52.1%), and Libo Kemkem (39.7%)^24,33^. These differences might have occurred from the fact that it has been believed VL is endemic mainly in the northwest parts of Ethiopia, and at the same time health posts out there are more well-developed to treat this particular case than the current study areas, and the communities in the northwest have better access for the information of treatment against VL cases.

In the current study, an overall 77.25% (309/400) of participants demonstrated good knowledge of CVL, with higher percentages of participants from the town of Ejaji 92% (184/200) than Weliso 62.5% (125/200). As a result, the results are better than those obtained from studies carried out in Tunisia; in the districts of Welkait, Western Tigray, Ethiopia; West Armachiho, Northwest Ethiopia; and Metema and West Armachiho, Northwest Ethiopia, where 46%, 58.71%, 21.1%, and 33.2% of the respondents, respectively, were knowledgeable^10,23,25,33^. The current study's findings, however, are less impressive than those of a study carried out in Addis Zemen, Northwest Ethiopia, where 89.4% of participants demonstrated understanding^22^.

Therefore, basically, the variability between the findings of knowledge of the current study and the previous ones might have been linked to factors such as the study's time frame, geographic locations, sample characteristics (age, education level, occupation), dog ownership, differences in research and surveillance activities focused on CVL and HVL, the endemicity of the disease in particular study areas, access to Health posts mainly focused on ZVL cases, awareness made by Veterinary and Medical professionals, level of participants' education and perception of information, individuals' access to better information from a wide range of sources, and due to the lack of One Health concept in ZVL assessment.

According to the results of the current study, the study town (Ejaji) and dog ownership were each (*p* = 0.001) significantly associated with the participants' good knowledge of CVL. Hence, the current study's risk factors of knowledge differed from studies conducted in the Districts of Metema and West Armachiho; West Armaciho, Northwest Ethiopia; endemic areas Northwest Ethiopia; and Welkait, Western Tigray, Ethiopia, where (age, income, history of VL, and having health information), (sex and residence), [the location (rural)], (sex, educational status, and histo-ry of travel), respectively, were found to be significantly associated with good knowledge of participants^23, 25, 29, 33^.

Therefore, since, the area is malaria endemic, the ongoing mosquito control programs in the Elu Gelan District (Ejaji is the headquarters) to combat malaria and due to the health promotion activities of Elu Gelan District Veterinary and Medical professionals in this career, as well as the experience and familiarity of dog owners with the diseases of the dogs, might have contributed to the significant association of study towns and dog ownership with the participants' good knowledge in the current study.

### Attitudes of the respondents

In the present investigation, the perception of CVL as a health concern among participants was reported at 78.25%. This percentage surpasses the results of a previous survey conducted in Addis Zemen town, Northwest Ethiopia, where only 53.1% of participants acknowledged VL as a health issue in both Addis Zemen town and the neighboring Kebeles^22^. However, the current finding falls short of the findings from the Welkait District in Western Tigray, Ethiopia, where a higher proportion of participants, specifically 84.09%, recognized VL as a health problem for the local population^23^. In fact, it is difficult to assess the results of attitudes of different studies. However, it is believed that the state of mind of every participant matters in perceiving any information regarding such zoonotic diseases.

Approximately 81.25% of participants in the present study expressed their concern about contracting any form of leishmaniasis. This finding is in close agreement with the results obtained from a study conducted in Welkait District, Western Tigray, Ethiopia, where 77.65% of participants believed they were at risk of acquiring the disease^23^. Around 72% of participants agreed that VL is a treatable illness which is higher than the findings reported in studies conducted in the Northwest Ethiopian districts of Libo Kemkem and Armachiho, where only 44.5% of participants in each district believed that the condition could be completely cured^24,25^. However, it is lower than the findings of studies conducted in Welkait District, Western Tigray, Ethiopia, and Addis Zemen town of Northwest Ethiopia, where the majority of participants, specifically 88.26% and 86.4% respectively, considered VL to be a curable disease^22,23^. These differences are also believed to occur because of individuals' access to better information from a wide range of sources.

In the current study, 42% of participants chose CVL medicine obtained from a health center. The outcome is lower than research done in Tunisia; Addis Zemen town; the Districts of West Armachiho, Northwest Ethiopia; Welkait, Western Tigray; Sudan; and a highly endemic rural area of India, where 78%, 94.4%, 62.3%, 97.35%, 76.8%, and 95% of participants, respectively, sought medication from health centers as soon as they and their dogs felt ill^10,22,23,25,31,34^. Besides, 12% of participants mentioned using dog collars and anti-leishmanial medications to treat CVL. The finding differs from a survey done in the Libo Kemkem District of Northwest Ethiopia, where 39.7% of participants preferred certain medications to treat VL^24^. Furthermore, 14.5% of participants expressed their belief in community involvement as a means of preventing and controlling CVL. This finding differs from those of studies carried out in Addis Zemen Town, Northwest Ethiopia, and Welkait District, Western Tigray, Ethiopia, where roughly 80% and 73.11% of participants, respectively, thought that VL could be controlled through community participation^22,23^. Basically, these differences in attitude results seem to occur mainly from a lack of uniform awareness-raising activities and access to better medication against such deadly vector-borne zoonotic diseases based on the One Health concept in every endemic area.

Therefore, when the participants’ overall attitude is considered, the majority of them (242/400, 60.5%) had a favorable attitude. Therefore, the current study's findings are better than those of studies carried out in the Northwest Ethiopian Districts of Metema and West Armachiho and West Armachiho, where 30.2% and 53.6% of participants, respectively, had a favorable attitude^25,33^. However, it was lower than a study conducted in Addis Zemen town, Northwest Ethio-pia, in that 87.1% of participants had a favorable attitude^22^.

The variation in attitudes of communities towards CVL between different studies in different countries can be attributed to several factors, including socioeconomic factors, the culture of society, the difference in regional disease burden, the difference in public health initiatives, the individual's willingness and level of perception of information regarding ZVL, government policies, and regulations, prioritization of extensive research and effective communication of information regarding such fatal zoonotic diseases tend to exhibit differences of awareness and favorable attitudes toward the disease.

According to the results of the current study, study towns, and dog ownership were each significantly (*p* = 0.001) associated with participants' favorable attitudes. Hence, the current study's risk factors of attitude results were contrasted with a study conducted in the Districts of West Armachiho; Metema, and West Armachiho, Northwest Ethiopia; where (sex, education and residence), and (the number of visits to the farm area, history of VL, health information, and kn-owledge), respectively, were significantly associated with a favorable attitude.

Therefore, since, the area is malaria endemic, the ongoing mosquito control programs in the Elu Gelan District (Ejaji is the headquarters) to combat malaria and due to the health promotion activities of Elu Gelan District Veterinary and Medical professionals in this career, as well as the experience and familiarity of dog owners with the diseases of the dogs, might have contributed to the significant association of study towns and dog ownership with the participants' favorable attitudes in the current study.

### Practices of the respondents

About 87.25% of participants in the current study were aware of the local custom of sleeping outdoors. In studies conducted in various regions of Northwest Ethiopia, the percentage of participants who reported having family members who slept outside was indicated as 10.6%, 62.88%, and 63%^22,23,30^. Also, 63.89% of participants reported that at least one member of their family frequently sleeps in corrals or pens for animals, on farms, or in tree shade. This finding is lower than those of studies carried out in Addis Zemen Town, Northwest Ethiopia, andRural Communities of Amhara State, Northwest Ethiopia, where 81.3% and 80% of participants, respectively, reported having observed outdoor and close-by animals sleeping^22,30^. However, it was higher than in research done in Welkait District, Western Tigray, Ethiopia, where 44.32% of the participants practiced sleeping next to animals^23^. When we compare the practical habits of individual participants, it is better to focus on the culture of the study areas or shortly the culture of the study areas greatly matters over the practical habits of participants.

In the current study areas, approximately 61.5% of participants reported using repellents during the humid seasons to avoid getting bitten by sandflies. This statistic was meaningfully greater than that of a study done in Welkait District, Western Tigray, Ethiopia, where 33.33% of the participants used repellents to shield themselves from any biting flies. As a similar precaution against sandfly bites during the humid seasons, 45.53% of participants in the current study areas employed natural wood smoke. This result was lower than a survey carried out in Welkait District, Western Tigray, Ethiopia, where 61.74% of the participants smoked chopped plant parts to ward off any biting insects^23^.

Additionally, Malathion and canned pesticide use rates in the current study areas are 22.76% and 15.85%, respectively. These findings surpass those of studies done in the Districts of Metema and West Armachiho, in Northwest Ethiopia, and the Welkait District, Western Tigray, Ethiopia, where 12.11% of respondents use spray insecticides and 94.5% of respondents in Welkait have never used insecticide spray on their homes or the area around them^23,33^. Moreover, 5% of respondents in the current study areas were found to use additional methods, such as burning dung and powder insecticides. This finding was consistent with research done in Addis Zemen, Northwest Ethiopia, where just 3.6% of respondents use dichloro-diphenyl-trichloroethane^22^. In fact, it is also difficult to compare individuals’ interests in using different drugs of choice. However, the socioeconomic statuses of the study areas have a great impact on drug choices.

The vast majority of respondents (64.5%) in the current study areas regularly clean their homes’ walls. This outcome was better than that of a survey carried out in the Metema and West Armachiho Districts, Northwest Ethiopia, where 35.4% of respondents cleaned their living space to prevent VL^33^. Also, 68.75% of respondents in the current study areas said that CVL can be controlled. Perhaps the quality of living and level of education of the participants might have jammed on the differences in these regards.

The current study showed that overall, 59.25% (237/400) of participants generally had good practices of CVL. Of these 59% (119/200) were from the towns of Weliso and 59.5% (119/200) were from Ejaji. These findings closely match those from rural areas of Nepal, where 58% of villagers in Titaria had good practices Still, they are lower than those from Haraincha, where 36.8% of villagers had good practices^35^, and higher than those from rural areas of India's Bihar state, where 23.9% of participants had good practices^32^. Once more, the current results are much better than a study carried out in the West Armachiho District, Northwest Ethiopia, where 14.9% of the participants had good practices on VL^25^.

According to the results of the current study, dog ownership (*p* $=$ 0.001), education level of Di-ploma (*p* = 0.019), degree and above (*p* = 0.018), marital status of divorced and widowed (P = 0.012), and ethnicity of Oromo (*p* = 0.013) are strongly associated with good practice towards CVL.

Therefore, in the current study, the significant association of the aforementioned risk factors with good practice of the study participants could potentially be attributed to the dog owner's experience and knowledge about canine diseases. Their familiarity with these diseases likely contributes to their adherence to preventive measures. Also, individuals with higher levels of education are more likely to have access to a wider range of information sources, including public and social media platforms, as well as school-based awareness initiatives. This increased exposure to information enabled them to gain a better understanding of effective techniques for preventing and controlling vector-borne diseases like VL. In addition, the association between good practice and widowed or divorced individuals could be linked to their utilization of dogs as companion animals to combat feelings of loneliness and isolation. Since the current study was conducted in the Oromia regional state, a large number of ethnic Oromo individuals were participated and found to be associated with good practices.

To sum up, the variability between the findings of the current study and the previous ones might be linked to a lack of community health education based on the One Health concept, differences in research and surveillance activities focused on CVL and HVL, the lack of uniformity and coordination to tackle ZVL in the spirit of the One Health concept, lack of community awareness made by Veterinary and Medical professionals, the endemicity of the disease, environmental factors including climate and vector abundance, the disease treatment, prevention, and control program of different regions, willingness of the study participants to take part in KAP studies of such zoonotic diseases, the socioeconomic status of the different areas, level of participants' education and perception of information, the individual's knowledge and access to better information from different sources (public and social media, awareness-raising activities in schools), culture and the people’s tradition to ask and seek information, which enabled people to gain a better understanding about such fatal vector-borne zoonotic diseases influence on the awareness level of the community about ZVL.

### Limitations of the research

In fact, it is difficult to give clear, specific, and possible explanations for any comparisons of the current findings with previous KAP studies designed to assess the HVL. In Ethiopia, the KAP component of CVL has received limited research focus and attention. There is a lack of comprehensive understanding of CVL in Ethiopia as a whole, particularly in the specific study areas. Consequently, the current study relied mainly on discussing the findings by referencing KAP questionnaire surveys conducted on HVL to compensate for the existing data gaps. Self-reported data, which might be skewed by participants' recall bias or social desirability bias, are frequently encountered in KAP investigations. As a result, the assessments of actual knowledge, attitudes, and practices linked to the targeted disease might be overestimated or underestimated. Therefore, results from the current KAP study might not be broadly applicable to other populations or locations and may not be generalizable due to the variability of study designs, and cultural, socioeconomic, and environmental factors among regions. The cross-sectional nature of the design may also limit our capacity to demonstrate causation between risk factors and results and to comprehend changes in knowledge, attitudes, and practices over time.

## Conclusions

Most respondents in the current study areas had generally good knowledge but moderate attitude and practices. However, participants from Ejaji town in particular fared far better in terms of knowledge and attitude than those from Weliso town. While study towns and dog ownership are strongly associated with good knowledge and a positive attitude toward CVL; dog ownership, level of education, marital status, and ethnicity are predictors of good practice toward CVL, in the current study areas. Therefore, it is crucial to undertake comprehensive community health education and awareness programs of ZVL and its vectors based on the One Health concept through various means.

## Data Availability

The data supporting the conclusions of this article are included within the article and anonymized data could be shared upon request to the corresponding author.

## Abbreviations

CVL: Canine visceral leishmaniasis
KAP: Knowledge, attitude, and practice
NTD: Neglected tropical disease
VL: Visceral leishmaniasis
CL: Cutaneous leishmaniasis
MCL: Mucocutaneous leishmaniasis
SNNPR: South Nations nationalities and people’s region
HVL: Human visceral leishmaniasis
M.A.S.L.: Meter above sea level
CSA: Central statistics agency
AOR: Adjusted odds ratio
CI: Confidence interval
COR: Crude odds ratio
ZVL: Zoonotic visceral leishmaniasis
